# Profiling lung adenocarcinoma by liquid biopsy: can one size fit all?

**DOI:** 10.1186/s12645-016-0023-8

**Published:** 2016-11-22

**Authors:** Harry W. Clifford, Amy P. Cassidy, Courtney Vaughn, Evaline S. Tsai, Bianka Seres, Nirmesh Patel, Hannah L. O’Neill, Emil Hewage, John W. Cassidy

**Affiliations:** 1 OneTest Diagnostics, Cambridge Applied Research, Future Business Centre, Cambridge, UK; 2 St. Edmund Hall, University of Oxford, Queen’s Lane, Oxford, UK; 3 NHS Greater Glasgow and Clyde, University of Glasgow, Glasgow, UK; 4 UNC School of Medicine, University of North Carolina, Chapel Hill, NC USA; 5 Peterhouse, University of Cambridge, Trumpington Street, Cambridge, UK; 6 Max Planck Institute for Biophysical Chemistry, Göttingen, Germany; 7 Division of Cancer Studies, King’s Health Partners AHSC, Faculty of Life Sciences and Medicine, King’s College London, London, UK; 8 Aberdeen Royal Infirmary, University of Aberdeen, Aberdeen, UK; 9 Queens’ College, University of Cambridge, Silver Street, Cambridge, UK; 10 Cancer Research UK Cambridge Institute, University of Cambridge, Cambridge, UK

**Keywords:** Lung adenocarcinoma, Cancer genomics, Mutation, Tumour suppressor, Oncogene, SNV, Circulating tumour DNA, ctDNA, Liquid biopsy

## Abstract

**Background:**

Cancer is first and foremost a disease of the genome. Specific genetic signatures within a tumour are prognostic of disease outcome, reflect subclonal architecture and intratumour heterogeneity, inform treatment choices and predict the emergence of resistance to targeted therapies. Minimally invasive liquid biopsies can give temporal resolution to a tumour’s genetic profile and allow the monitoring of treatment response through levels of circulating tumour DNA (ctDNA). However, the detection of ctDNA in repeated liquid biopsies is currently limited by economic and time constraints associated with targeted sequencing.

**Methods:**

Here we bioinformatically profile the mutational and copy number spectrum of The Cancer Genome Network’s lung adenocarcinoma dataset to uncover recurrently mutated genomic loci.

**Results:**

We build a panel of 400 hotspot mutations and show that the coverage extends to more than 80% of the dataset at a median depth of 8 mutations per patient. Additionally, we uncover several novel single-nucleotide variants present in more than 5% of patients, often in genes not commonly associated with lung adenocarcinoma.

**Conclusion:**

With further optimisation, this hotspot panel could allow molecular diagnostics laboratories to build curated primer banks for ‘off-the-shelf’ monitoring of ctDNA by droplet-based digital PCR or similar techniques, in a time- and cost-effective manner.

## Background

Cancer is a disease of the genome; one which is initiated by nanostructural perturbations in the structure and function of DNA (e.g. somatic mutations, epigenetic modifications, etc.) and driven by the sequential accumulation of these perturbations (Hanahan and Weinberg 2011). The study of genomic aberrations and the identification of somatic mutations that drive a particular malignancy are, therefore, fundamental to the understanding of tumour biology. In addition, targeted therapies developed to inhibit the growth of a tumour are almost exclusively stratified to patients harbouring specific mutational profiles (Huang et al. 2014). For example, cetuximab, an anti-epidermal growth factor receptor (EGFR) therapy, is only truly effective in patients with EGFR amplifications (Yang et al. 2013). Tumour genotype information is needed by clinicians on a per patient basis.

Resistance to targeted therapies often emerges during a treatment regimen. Pre-existing resistant populations in a treatment-naïve tumour and induced-resistant populations acquired de novo during therapy have both been described as mechanisms of resistance. Bhang and colleagues have recently traced the emergence of erlotinib resistance in a model of lung adenocarcinoma, identifying a pre-existing *MET*-amplified clonal population responsible for in vitro recurrence (Bhang et al. 2015). In a separate lung cancer model, Hata et al. showed that EGFR^T790M^ mutations could be acquired during navitoclax therapy and drive the inhibitor-resistant phenotype (Hata et al. 2016). Thus, including temporal resolution in cancer genomic information will better inform treatment decisions.

Because of the clinical importance of tumour genomics, it is unsurprising that the sequencing of tumour biopsies prior to, and during, treatment regimens has become commonplace over the past several years. However, spatial heterogeneity within a tumour can lead to an under-representation of intratumour heterogeneity and an inaccurate reporting of tumour genotypic information gleamed from punch biopsies (Sottoriva et al. 2013; de Bruin et al. 2014). Moreover, such biopsies are relatively invasive for solid tumours. Thus, many researchers and clinicians alike have turned to so-called ‘liquid biopsies’ in an attempt to identify circulating mutant tumour DNA (ctDNA) in a patient’s blood (Newman et al. 2014; Ma et al. 2015). By deep molecular characterisation of this ctDNA across multiple sequential biopsies, it is hoped that researchers and oncologists will gain a better picture of cancer’s genetic makeup and how this evolves over time, without the considerations associated with spatial heterogeneity.

Typically, profiling of ctDNA is achieved through deep or targeted amplicon sequencing (Newman et al. 2014). However, this approach is limited in terms of cost and throughput. For some of the more immediate clinical applications of ctDNA, such as tracking treatment response, temporal resolution of a tumour’s evolution may be as useful as a deep understanding of its molecular drivers. Thus, many approaches for sequential monitoring of ctDNA have focussed on high-throughput techniques such as droplet and digital PCR to trace individual mutations in a patient’s blood over time (Zheng et al. 2016). In this study, we sought to determine whether a panel of recurrently mutated genomic loci (hereafter ‘hotspots’) could be developed which would give suitable coverage over the entirety of the intertumour heterogeneity seen in human malignancies. As a test case, we focus on lung adenocarcinomas: a malignancy that is not well suited to typical punch biopsy techniques and that has substantial genomic heterogeneity amongst the clinical population.

## Results

### Lung adenocarcinomas are characterised by genomic aberrations in 23 driver genes

**Fig. 1:**
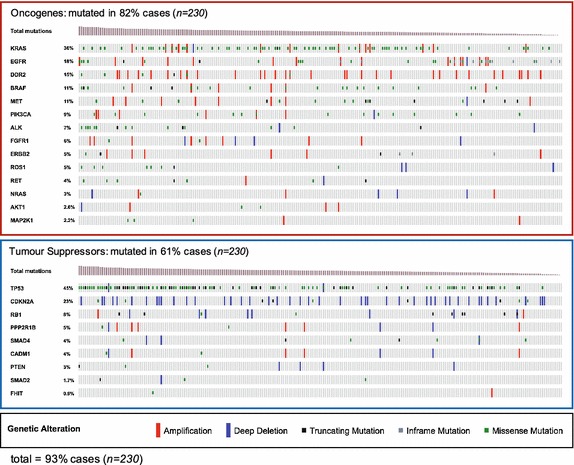
Waterfall plots of genetic alterations in The Cancer Genome Atlas lung adenocarcinoma dataset (*n* = 230). Each* column* represents an individual patient, colour-coded based on copy number aberration (amplification or deletion) and/or mutational state (truncating mutation, inframe mutation, missense mutation) across profiled oncogenes and suppressors frequently altered in lung adenocarcinoma. Columns are sorted based on total mutational burden. In total, 93% of patients in the dataset are covered by one or more genetic aberration in the 23 genes profiled

We first sought to profile the mutational landscape of lung adenocarcinomas, focussing on copy number aberrations (amplifications and deletions), single nucleotide variations (SNVs: non-synonymous, missense, and nonsense mutations), and frameshift mutations (truncating and inframe) across a panel of key driver genes. We identified a panel of 14 oncogenes previously reported to be of key importance in lung adenocarcinoma (Fig. 1, upper panel). Alterations in these genes are present in 82% of our test population of 230 patients (TCGA) (Network 2014). The landscape here is dominated by missense mutations in *KRAS* (present in >30% cases) and copy number gains primarily in *EGFR, DDR2, BRAF, MET* and *PIK3CA*. Importantly, targeted therapies have been developed for each of these driver genes. Aside from *KRAS*, SNVs are relatively evenly distributed across these driver oncogenes. Samples were sorted by overall mutational burden. Despite covering the vast majority of patients, our oncogene panel did not cover the bulk of a patients’ mutational burden, likely due to a high proportion of low-frequency ‘passenger’ mutations within the clinical population.

Next, we performed the same analysis on nine previously described tumour suppressor genes frequently altered in lung adenocarcinoma (Fig. 1, lower panel). Unsurprisingly, *TP53* was the most frequently mutated tumour suppressor gene with >40% of patients harbouring a missense or truncating mutation. *CDKN2A* was the next most frequently altered gene with >20% of patients carrying copy number losses. Altogether, the nine tumour suppressors profiled were altered in 61% of the 230 test cases.

In total, 93% of the 230 patients possessed at least one genomic aberration in our panel of 23 drivers, with >50% having alterations in two or more genes (a ‘depth’ of two per patient). Although the detection of copy number aberrations in ctDNA is possible (Bettegowda et al. 2014), we elected to focus the rest of the analysis on SNVs and frameshift mutations, which can be detected with greater confidence across a wider range of techniques.

### Hotspots in frequently mutated drivers are relatively rare

Most techniques aimed at detecting mutational events within a gene, such as digital droplet PCR or SNV array technologies, detect specific base-pair substitutions or frameshift mutations at a defined genomic locus rather than across the entire gene length. Despite the fact that over 30% of patients harbour a missense mutation in *KRAS*, many of these mutations could be missed without proper direction. Thus, it is important to identify specific hotspot loci within driver genes to create targeted panel.

**Fig. 2:**
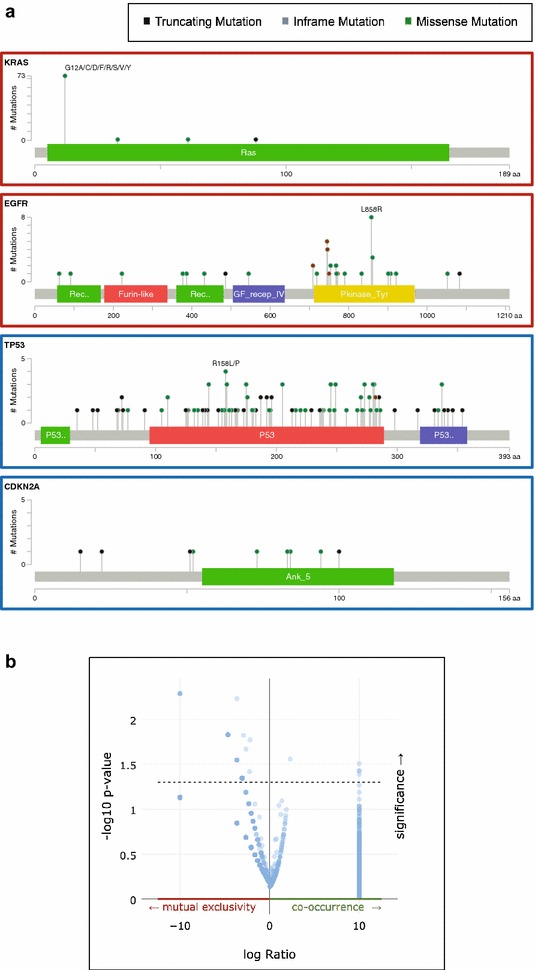
**a** Lollipop plots showing the distribution of mutations (colour coded as Fig. 1) across the protein-coding regions of the two most commonly mutated oncogenes (*KRAS* and *EGFR*) and tumour suppressors (*TP53* and *CDKN2A*).** b** Volcano plot showing tendency towards mutual exclusivity or co-occurrence of mutations in the 23 genes profiled in Fig. 1

We identified several such hotspot regions in recurrently mutated oncogenes (representative examples *KRAS* and *EGFR* in Fig. 2a), For example, 73 of the 75 SNVs in *KRAS* resulted in an amino acid substitution at position 12 in the Ras domain. Notably, 17% of *KRAS* mutant lung adenocarcinomas harbour the G12D substitution (glycine to aspartic acid at position 12) which confers a more invasive tumour phenotype and a reduced response to anti-EGFR targeted therapies (Gallegos Ruiz et al. 2007; DuPage et al. 2009). *EGFR*, the second most recurrently mutated oncogene in lung adenocarcinoma, showed a much more dispersed pattern of mutational events across its protein-coding domains. Missense mutations were preferentially localised to the phosphor-tyrosine kinase (Pkinase_Tyr) and eight resulted in an amino acid substitution from leucine to arginine at position 858 (L858R) (Network 2014; Zheng et al. 2016).

Profiling the recurrently mutated tumour suppressor genes, *TP53* and *ANK5* (Fig. 2a, lower panels) revealed a near even distribution of missense and truncating mutations. This supports the longstanding observation that tumour suppressor genes do not tend to have hotspot regions that confer a change in catalytic activity but rather tend to be truncated or deleted in late-stage malignancies. Indeed, the observation that tumour suppressors do not tend to have recurrent hotspot regions is the basis of the 20/20 rule often used to define tumour drivers (Vogelstein et al. 2013). Analysis of the tendency for mutations in our 23 driver genes to co-occur across multiple patients revealed the same pattern. Whilst a number of mutations do co-occur, the majority are mutually exclusive (Fig. 2b). Thus, it is likely that a panel of recurrently mutated regions in our 23 driver genes would not be enough to cover a substantial proportion of lung adenocarcinoma patients to a high depth.

### Genome-wide panels of recurrently mutated regions cover >80% patients

**Fig. 3:**
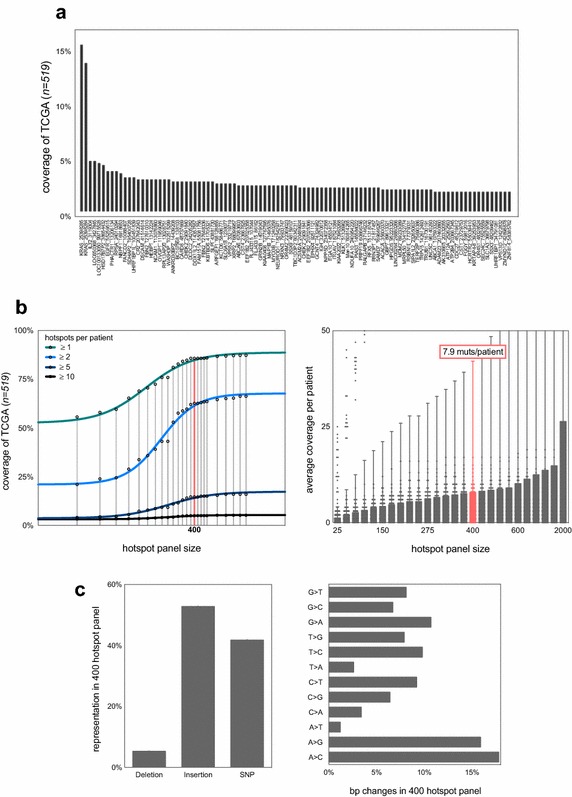
**a** 100 specific loci (“hotspots”) frequently mutated in the lung adenocarcinoma dataset (*n* = 519) ranked based on shared coverage across the dataset. Annotation is *geneID_genomic*-*start*-*location.* For example, a hotspot region in *KRAS* starting at 25398285 is mutated in >15% of the TCGA dataset (this is the G12D region depicted in Fig. 2a). **b** Correlation between the size of a hotspot mutational panel and coverage in the TCGA dataset at specific depths [≥1, 2, 5 or 10 mutations per patient (*left panel*) and mean depth ± standard deviation (*right panel*)]. A panel size of 400 hotspots is highlighted for ease of comparison. The top 400 most frequently mutated regions in the dataset cover >75% patients at a depth of one mutation and >50% at a depth of two mutations (*left panel*); with a mean coverage of 7.9 per patient (*right panel*). **c** The composition of this 400 hotspot panel. The panel is dominated by SNVs and insertions (*left panel*) and is relatively balanced in terms of basepair substitution (*right panel*)

Given that the lung adenocarcinoma driver gene panel would not provide sufficient coverage, we elected to identify recurrently mutated genomic loci in an unbiased, genome-wide screen. Called somatic mutations were downloaded from the TCGA data portal in mutation annotation format (MAF) and unique loci were identified and scored based on frequency and distribution across the whole dataset (*n* = 519). The top 100 recurrently mutated loci in TCGA lung adenocarcinomas (Fig. 3a) had a median coverage of 3% (i.e. mutated in 3% of TCGA patients). Amongst these recurrent hotspots were two loci in the *KRAS* gene identified in Fig. 2, alongside novel mutations such as in *IL32* (5.2% coverage) and *RPSA* (4.2% coverage). *IL32* encodes a cytokine that is up-regulated in lung adenocarcinomas and is correlated with lymph node metastasis (Sorrentino and Di Carlo 2009). *RPSA* encodes a ribosomal entry protein known to be up-regulated in lung adenocarcinomas but to an unknown end (Wu et al. 2013).

As our panel of 100 hotspots only covered 59% of TCGA patients, we examined the panel size needed to cover a majority of patients at a relatively high depth. Figure 3b shows the correlation between size of mutational panel and overall coverage of the dataset for four different representative depths. We start to see diminishing returns in coverage at a hotspot panel size of 1000 mutations. Therefore, covering the majority of patients at a depth greater than 10 mutations is unlikely. This highlights the intertumour heterogeneity seen between patients with lung adenocarcinoma (Zhang et al. 2014). However, a 400-mutation panel gives a median coverage of 7.9 mutations per patient (Fig. 3b, right panel) with 82.8% patients covered by at least one mutation and 57.6% of patients covered by two or more mutations. The 400-mutation panel is dominated by insertions and SNVs (Fig. 3c, left) and is balanced in terms of specific base-pair changes (Fig. 3c, right). Although the 400-mutation panel does not cover the entirety of TCGA lung adenocarcinoma patients, its scale is feasible for a molecular diagnostics lab. Thus, probes for these 400 mutations could be optimised for off-the-shelf use in clinics—with the addition of more targeted probes for specific patients.

## 400 SNV hotspot panel covers >55% of 183 patients in Broad validation set

**Fig. 4:**
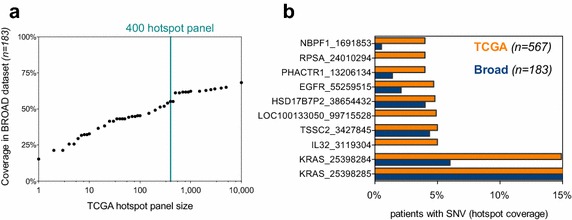
**a** Coverage of TCGA hotspot panels (of different sizes) in a validation dataset from the Broad Institute (*n* = 183). A panel of 400 hotspots delineated in the TCGA dataset is highlighted for ease of interpretation—the 400 most frequent mutations in TCGA cover 55.49% of Broad patients. Even with the 10,000 most frequent mutations in TCGA, only 68% of Broad patients are covered.** b** The ten most frequent mutations in TCGA are presented with their coverage of TCGA and Broad patients. Three of the top 10 most frequent mutations in TCGA are not seen at all in the Broad dataset

To validate our panel of 400 mutations from the TCGA dataset, we analysed the most frequent hotspot SNVs found in 183 patients sequenced by the Broad Institute (Imielinski et al. 2012). The most frequent mutations in each dataset were relatively common, validating our approach. For example, a panel of 10 common hotspots from TCGA covered 32.7% of Broad patients at a depth of at least one mutation per patient (Fig. 4a). However, our panel of 400 hotspots from TCGA only covered 55% of patients in the validation dataset. Indeed, extending the panel size to the most common 10,000 hotspots in TCGA only allowed for coverage of 68% of the 183 Broad patients. These data suggest the need for further sequencing of lung adenocarcinoma patients to better understand the prevalence of less-frequent mutations. Interestingly, there is a marked difference in the prevalence of the 10 most frequent mutations in TCGA between the two datasets (Fig. 4b). SNVs at IL32 (starting at 3119304), LOC100133050 (starting at 99715528) and RPSA (starting at 24010294) were not present at all in the Broad dataset despite high prevalence in TCGA. These observations could be a feature of different filtering techniques in mutation calling in each study, or small sample size in the Broad dataset.

## Discussion

Over the past several years, renewed effort in cancer research has yielded a myriad of molecular drivers of and contributors to tumour progression. Alongside the most often cited contributors, there are changes in stromal cell infiltrates (Kalluri and Zeisberg 2006), alterations in receptor prevalence or cell signalling (O’Neill et al. 2016), and nanotopographical changes to the cancer cell’s niche (Cassidy 2014; Cassidy et al. 2014). However, cancer is fundamentally a disease of the genome and only by understanding the patterns of clonal dynamics and evolution of genomic clones will the disease be fully understood.

As the need for accurate and temporally specific genomic information makes its way into the clinical setting, we must adopt new methodologies of profiling a tumour’s genome in a non-invasive and low-cost manner. Analysis of ctDNA has shown much promise in this regard, being used in many pioneering studies for monitoring treatment response, predicting relapse, and profiling intratumour heterogeneity (Bettegowda et al. 2014; Ma et al. 2015; Zheng et al. 2016). However, analysis of ctDNA is often initially based on targeted sequencing, which is both expensive and time consuming. Typically, specific primers can be designed after initial sequencing and ctDNA levels in the blood can be followed by less-demanding techniques, such as droplet digital PCR (Zheng et al. 2016). In this study, we set out to identify a panel of recurrent mutations in lung adenocarcinoma that would cover the majority of patients. Primers could then be designed and optimised for this panel ready for ‘off-the-shelf’ use in molecular diagnostic laboratories.

Lung adenocarcinoma is particularly heterogeneous and, even with a panel of 400 recurrent hotspots, coverage of 1× was only possible in ~80% of patients (Fig. 3b). This is particularly problematic as many of these mutations are likely passengers and therefore not necessarily clonal to the whole tumour. Thus, with a coverage of 1× we could not be sure that ctDNA levels were truly representative of the tumour bulk as a whole. However, this panel could be substantially refined in the future given the prevalence of recurrent copy number aberrations in driver genes seen in Fig. 1, and the recurrent promoter methylation in lung cancer (Belinsky 2004) which is recapitulated in ctDNA (Mishima et al. 2015; Warton et al. 2016). Care should also be taken to include likely ‘truncal’ genomic aberrations common to the tumour as a whole and not restricted to minor subclonal populations. Differences in TCGA and Broad datasets (Fig. 4) reflect tumour heterogeneity in lung adenocarcinomas and suggest that recurrently methylated CpG sites may also require inclusion in such panels. Although if such efforts relied on bisulfide conversion of CpG islands, we may see a loss of resolution for “C to T” SNVs at these sites.

The need for rapid identification of ctDNA in the time- and cost-constrained environment of clinical oncology is clear, and lung adenocarcinoma is of particular interest due to the difficulty in collecting recurrent solid biopsies. Our study aimed to identify a targeted hotspot panel for lung adenocarcinoma. We described mutation patterns in known genetic drivers of lung adenocarcinoma and profiled genome-wide recurrently mutated loci. Moreover, this work has identified several novel recurrent mutations in genes not typically associated with lung adenocarcinoma, which are each present in a significant subset of TCGA lung adenocarcinoma patients (Fig. 3a, e.g. *IL32, LOC650368, HSD17B7P2* and *RPSA*). Whilst our panels were informative, they did not provide sufficient coverage and depth to be clinically useful. Future work should refine our initial panel to include recurrent copy number aberrations and hyper-methylated promoter regions.

## Conclusions

ctDNA shows great promise for low-invasive serial monitoring of tumour burden and heterogeneity through treatment cycles. However, current ctDNA detection techniques rely on next-generation sequencing which is time consuming, expensive and requires bioinformatics expertise and access to specialist sequencing facilities. Tracing ctDNA through serial biopsy is better suited to high-throughput and low-cost techniques such as digital droplet PCR. In this scenario, a molecular diagnostics laboratory would first deeply sequence a patients’ ctDNA and then design primers for subsequent digital droplet PCR. In this study, we sought to define a panel of common hotspot mutations in lung adenocarcinoma to allow molecular diagnostic laboratories to design and optimise primers to cover the majority of patients. Although our 400-hotspot panel showed good coverage and depth in the TCGA dataset, all patients could not be covered. The difficulties in finding hotspots common to all patients reflect the profound intertumour heterogeneity seen in all cancers (Cassidy and Bruna 2016) and in particular lung adenocarcinomas. Further work is needed to optimise the panel design prior to use in the clinic, alongside continued collection of whole genome sequencing data from lung adenocarcinoma patients. Beyond mutations, efforts should be made to include recurrently methylated CpGs and copy number aberrations in such panels.

## Methods

Primary mutational analysis was carried out using cBioPortal (cbioportal.org) (Cerami et al. 2012; Gao et al. 2013). Lollipops were constructed using the R package ‘lollipops’ (github.com/pbnjay/lollipops), with pathway data obtained from Cytoscape 3.2.1 (cytoscape.org) (Lopes et al. 2010). Called somatic mutations (SNVs) and clinical metadata were downloaded from the TCGA Data Portal (tcga-data.nci.nih.gov) (Network 2014). Validation dataset from the Broad Institute was downloaded from dbGAP (Imielinski et al. 2012). Mutation annotation format (MAF) files were manipulated in R Studio (Mac) 0.99.484 (rstudio.com). Combined data were analysed in Microsoft Excel (Mac 14.4.3) and R Studio with results plotted in GraphPad Prism 6 (Mac) and R (3.3.1 Unix; r-project.org).

## Abbreviations

ctDNA: circulating tumour DNA
DNA: deoxyribonucleic acid
EGFR: epidermal growth factor receptor
MAF: mutation annotation format
MET: hepatocyte growth factor receptor
PCR: polymerase chain reaction
SNV: single nucleotide variation
TCGA: the cancer genome atlas
